# Metabolomics of Dietary Intake of Total, Animal, and Plant Protein: Results from the Atherosclerosis Risk in Communities (ARIC) Study

**DOI:** 10.1016/j.cdnut.2023.100067

**Published:** 2023-03-24

**Authors:** Lauren Bernard, Jingsha Chen, Hyunju Kim, Kari E. Wong, Lyn M. Steffen, Bing Yu, Eric Boerwinkle, Casey M. Rebholz

**Affiliations:** 1 Department of Epidemiology, Johns Hopkins Bloomberg School of Public Health, Baltimore, MD, USA; 2 Division of Nephrology, Department of Medicine, Johns Hopkins University, Baltimore, MD, USA; 3 Metabolon, Research Triangle Park, Morrisville, NC, USA; 4 Division of Epidemiology and Community Health, University of Minnesota School of Public Health, Minneapolis, MN, USA; 5 Department of Epidemiology, Human Genetics, and Environmental Sciences, University of Texas Health Science Center at Houston School of Public Health, Houston, TX, USA; 6 Human Genome Sequencing Center, Baylor Colleague of Medicine, Houston, TX, USA

**Keywords:** animal protein, dietary protein, metabolomics, nutrition, plant protein, total protein

## Abstract

**Background:**

Dietary consumption has traditionally been studied through food intake questionnaires. Metabolomics can be used to identify blood markers of dietary protein that may complement existing dietary assessment tools.

**Objectives:**

We aimed to identify associations between 3 dietary protein sources (total protein, animal protein, and plant protein) and serum metabolites using data from the Atherosclerosis Risk in Communities Study.

**Methods:**

Participants’ dietary protein intake was derived from a food frequency questionnaire administered by an interviewer, and fasting serum samples were collected at study visit 1 (1987–1989). Untargeted metabolomic profiling was performed in 2 subgroups (subgroup 1: *n* = 1842; subgroup 2: *n* = 2072). Multivariable linear regression models were used to assess associations between 3 dietary protein sources and 360 metabolites, adjusting for demographic factors and other participant characteristics. Analyses were performed separately within each subgroup and meta-analyzed with fixed-effects models.

**Results:**

In this study of 3914 middle-aged adults, the mean (SD) age was 54 (6) y, 60% were women, and 61% were Black. We identified 41 metabolites significantly associated with dietary protein intake. Twenty-six metabolite associations overlapped between total protein and animal protein, such as pyroglutamine, creatine, 3-methylhistidine, and 3-carboxy-4-methyl-5-propyl-2-furanpropanoic acid. Plant protein was uniquely associated with 11 metabolites, such as tryptophan betaine, 4-vinylphenol sulfate, *N*-δ-acetylornithine, and pipecolate.

**Conclusions:**

The results of 17 of the 41 metabolites (41%) were consistent with those of previous nutritional metabolomic studies and specific protein-rich food items. We discovered 24 metabolites that had not been previously associated with dietary protein intake. These results enhance the validity of candidate markers of dietary protein intake and introduce novel metabolomic markers of dietary protein intake.

## Introduction

Dietary intake of protein in United States adults is high and continues to increase over time [1]. Recent research has reported associations between dietary intake of total protein, animal protein, and plant protein and clinical outcomes such as chronic kidney disease, ischemic heart disease, stroke, and all-cause mortality [[2], [3], [4], [5]]. Although a higher intake of animal protein generally has been associated with a higher risk of these outcomes, a higher intake of plant protein has been associated with a lower risk of incident chronic kidney disease, ischemic heart disease, stroke, and all-cause mortality [[2], [3], [4], [5]]. Because each of these protein types has distinct relationships with clinical outcomes, interest has developed around improving assessment of dietary intake of specific protein sources.

Traditionally, dietary intake has been assessed through self-reported methods, such as 24-h recalls and food diaries. These assessment methods are prone to misreporting (e.g., recall bias) and measurement error (e.g., underestimation) [6,7]. For dietary intake of protein, 24-h urine nitrogen is an established biomarker [8]. However, it is burdensome for participants to collect urine over a 24-h period. Thus, new blood biomarkers are needed. One new approach is nutritional metabolomics, which studies small molecules in biofluids in relation to dietary intake [9].

To date, however, nutritional metabolomics has been sparsely applied to identify biomarkers of different sources of dietary protein (e.g., animal protein) [[10], [11], [12]]. Of the few studies that exist, only 1 study has identified plasma metabolites associated with plant protein intake [11]. More work is needed to identify biomarkers of dietary intake of total protein, animal protein, and plant protein. Broader metabolomic platforms need to be leveraged across larger samples to maximize the potential for biomarker discovery of dietary protein.

The overarching goal of this study was to improve dietary assessment with the discovery of objective biomarkers of dietary protein through the use of serum metabolomic profiling in 2 distinct samples of middle-aged adults. Compared with animal protein, we hypothesized that plant protein would have a distinct metabolomic profile given that dietary sources of plant protein have been previously reported to have unique metabolic signatures with fewer essential amino acids [13].

## Methods

### Study population and design

The Atherosclerosis Risk in Communities (ARIC) study is a prospective cohort study designed to investigate the causes and clinical outcomes of atherosclerosis. Beginning in 1987, the ARIC study enrolled 15,792 individuals from four United States communities (Forsyth County, NC; Jackson, MS; Minneapolis suburbs, MN; and Washington County, MD). A detailed description of the ARIC study design and methods have been previously published [14]. Institutional review boards (IRBs) at each field center approved of the ARIC study, and participants have given written informed consent at each study visit. This study received IRB approval from Johns Hopkins Bloomberg School of Public Health (IRB00012998; IRB00009957; IRB00011012). Procedures complied with the tenets of the Declaration of Helsinki.

Fasting serum samples were collected at visit 1 (1987–1989) and stored at −80°C until metabolomic profiling could be performed. Profiling was performed in 2 analytic subgroups in 2010 (subgroup 1) and 2014 (subgroup 2). Subgroup 1 included 1977 randomly selected Black participants from the Jackson, Mississippi field center, whereas subgroup 2 comprised a nonoverlapping set of 2055 participants from all 4 field centers. For this analysis, only participants with available metabolomic data were assessed for eligibility (*n* = 4032). Of the 4032 participants in subgroups 1 and 2, we excluded participants with missing values for covariates: BMI (*n* = 4), total energy intake (*n* = 59), smoking status (*n* = 5), physical activity (*n* = 15), education status (*n* = 5), alcohol consumption (*n* = 30), and specific dietary factors (i.e., total fruit, whole grains, and refined grains) (*n* = 0) (Supplemental Figure 1). We also examined 2 additional exclusions for participants missing protein intake information or unrealistic energy intake (defined as <600 kcal or >4200 kcal for men and <500 kcal or >3600 kcal for women), but these criteria did not result in any participants being excluded. Finally, 3914 participants were included in this analysis.

### Assessment of dietary protein intake

Dietary protein intake was assessed at visit 1 using a semiquantitative, 66-item food frequency questionnaire (FFQ) adapted from the Willett questionnaire [15]. The 66-item FFQ has been previously found to have high reproducibility and validity in a subset of 418 ARIC study participants [16]. To ascertain dietary intake, interviewers asked participants to report how often they consumed each food item of a specific serving size on average during the last year. Participants were provided with 9 frequency options, ranging from almost never to >6 servings/d. Protein–related FFQ items were used to create 3 exposure categories: total protein, animal protein, and plant protein (in grams). Similar to previous analyses in the ARIC study, plant protein was defined as the difference between total protein and animal protein [17,18].

### Assessment of metabolites

Fasting serum samples from visit 1 were analyzed by Metabolon, Inc., using an untargeted, gas chromatography/mass spectrometry and liquid chromatography/mass spectrometry-based protocol [19,20]. Metabolites were identified using a 2-tiered verification system. Tier 1 metabolites were compared with known reference standards and shared 2 orthogonal measurements with the standard. Tier 2 metabolites did not have a known reference available, but they were identified based on physiochemical properties or spectral similarities. Metabolites in tier 2 are represented with an asterisk in tables and figures.

Metabolomic assessment and data cleaning was performed using the same methodology as previous ARIC metabolomic studies [21,22]. Metabolites were rescaled in each sample to a median of 1 and log_2_-transformed. Metabolites were excluded if log-scale variance was low (i.e., <0.01), and values were capped at 5 SDs above the mean. After participant exclusion criteria were applied, metabolites were excluded if more than 80% of the analytic sample had missing values. For the primary analysis, metabolites not available in both samples (*n* = 367) were excluded. A total of 360 known metabolites were included in the primary analysis. All known nondrug metabolites with missing values were imputed to the minimum value of each metabolite. Secondary analyses of metabolites that met inclusion criteria in only one subgroup of participants included 2 metabolites (4-acetaminophen sulfate and hydrochlorothiazide) in subgroup 1 and 365 metabolites in subgroup 2. The discrepancy derives from improved metabolite identification that occurred by the time serum metabolites were measured in subgroup 2.

### Assessment of covariates

At visit 1, demographic and background characteristics were collected from interviewer-administered questionnaires. Age, total energy intake, physical activity, alcohol consumption, and specific dietary factors (total fruit, whole grains, and refined grains) were modeled as continuous variables. Physical activity was calculated as a score from 1 to 5, reflecting sport during leisure time, which incorporated intensity, time, proportion of year, and frequency, in addition to activity relative to peers and sweat frequency. Alcohol consumption included amount of beer, wine, and hard liquor in grams per week. To identify metabolites specific to total protein, animal protein, and plant protein, we calculated intake of several dietary factors and adjusted for these dietary factors as covariates. Total fruit intake (e.g., apples, pears, orange, peach, apricot, plum, bananas, and grapefruit), whole grains (e.g., grain bread and hot cereal), and refined grains (e.g., pie, donut, biscuit, pastry, cake, cookie, white bread, rice, and cold cereal) were estimated from the FFQ in units of servings per day. Sex, race, study center, cigarette smoking status, and education were modeled as categorical variables.

BMI was calculated from weight measured using a calibrated scale and height. Creatinine concentration was measured in serum at visit 1 with the modified kinetic Jaffe method. Then, the estimated glomerular filtration rate was estimated using the 2021 Chronic Kidney Disease Epidemiology race-free equation based on creatinine concentration [23].

### Statistical analysis

Descriptive statistics were used to describe the analytic sample overall and according to subgroup, and differences between subgroups were tested using χ^2^ tests for categorical variables and *t* tests for continuous variables. We used multivariable linear regression models to estimate cross-sectional associations between dietary protein sources and serum metabolites. Our model adjusted for age, sex, BMI, total energy intake, estimated glomerular filtration rate, smoking status, physical activity, education, alcohol consumption, total fruit intake, whole grains, and refined grains. We adjusted for alcohol consumption based on a previous study that demonstrated an effect of alcohol on the metabolome [24]. We adjusted for dietary intake of fruit, whole grains, and refined grains because they represent the other major nonprotein food groups. For subgroup 2, the model included two additional covariates, race and study center, because multiple race groups and study centers were represented in subgroup 2. Estimates were generated within each subgroup and meta-analyzed with a fixed-effects model. Bonferroni correction was used to correct for multiple comparisons. For the primary analysis of metabolites in both samples, our statistical significance threshold was *P* = 0.05/(360 metabolites × 3 protein sources) = 4.6 × 10^−5^. For the secondary analyses, our significance threshold in subgroup 1 was *P* = 0.05/(2 metabolites measured in sample 1 only × 3 protein sources) = 0.008 and in subgroup 2 was *P* = 0.05/(365 metabolites measured in sample 2 only × 3 protein sources) = 4.6 × 10^−5^. Analyses were conducted using Stata version 17 (StataCorp) and R version 4.1.2.

## Results

In the overall sample of 3914 participants, the mean age was 54 y, 60% were women, and 61% were Black (Table 1). One-third of the participants reported some college education. Subgroup 1 consisted exclusively of Black participants, whereas 27% of participants in subgroup 2 were Black (*P* < 0.001). There were slightly more women in subgroup 1 (64%) than those in subgroup 2 (57%) (*P* < 0.001). Total protein accounted for 18% of total energy intake, and approximately one-quarter of total protein intake was derived from plant protein sources. The average dietary intake of total, animal, and plant protein was similar across the two subgroups.

**TABLE 1:** Baseline characteristics of the ARIC study participants^1^

Characteristics	All subjects (*n=* 3914)	Subgroup 1 (*n=* 1842)	Subgroup 2 (*n=* 2072)	*P*
Age (y)	54.1 ± 5.8	53.4 ± 5.7	54.8 ± 5.7	<0.001
Female sex	2359 (60.3)	1184 (64.3)	1175 (56.7)	<0.001
Black	2398 (61.3)	1842 (100)	556 (26.8)	<0.001
Study site				<0.001
Forsyth County, NC	589 (15.1)	0 (0)	589 (28.4)	
Jackson, MS	2269 (58.0)	1842 (100)	427 (20.6)	
Minneapolis suburbs, MN	522 (13.3)	0 (0)	522 (25.2)	
Washington County, MD	534 (13.6)	0 (0)	534 (25.8)	
BMI (kg/m^2^)	28.7 ± 5.8	29.6 ± 6.0	28.0 ± 5.5	<0.001
Total energy intake (kcal/d)	1619.6 ± 617.2	1578.9 ± 621.8	1655.7 ± 611	<0.001
Estimated glomerular filtration rate (mL/min/1.73 m)	100.4 ± 14.8	100.3 ± 15.4	100.4 ± 14.3	0.79
Smoking status				<0.001
Current smoker	1077 (27.5)	519 (28.2)	558 (26.9)	
Former smoker	1085 (27.7)	417 (22.6)	668 (32.2)	
Never smoker	1752 (44.8)	906 (49.2)	846 (40.8)	
Education level				<0.001
Less than high school	1262 (32.2)	750 (40.7)	512 (24.7)	
High school or vocational school	1346 (34.4)	516 (28.0)	830 (40.1)	
Some college or more	1306 (33.4)	576 (31.3)	730 (35.2)	
Alcohol consumption (g/wk)	36.4 ± 94.6	31.9 ± 100.5	40.5 ± 88.9	0.005
Physical activity	2.3 ± 0.8	2.1 ± 0.7	2.4 ± 0.8	<0.001
Total protein intake (% of total energy)	17.9 ± 4.2	18.1 ± 4.3	17.8 ± 4.1	0.09
Animal protein intake (% of total energy)	13.6 ± 4.3	13.9 ± 4.4	13.4 ± 4.2	<0.001
Plant protein intake (% of total energy)	4.3 ± 1.2	4.2 ± 1.1	4.4 ± 1.3	<0.001

ARIC, Atherosclerosis Risk in Communities.

1 Values are mean ± SD or n (%). Differences in characteristics between the subgroups were tested using χ-tests for categorical variables and *t* tests for continuous variables.

**FIGURE 1:**
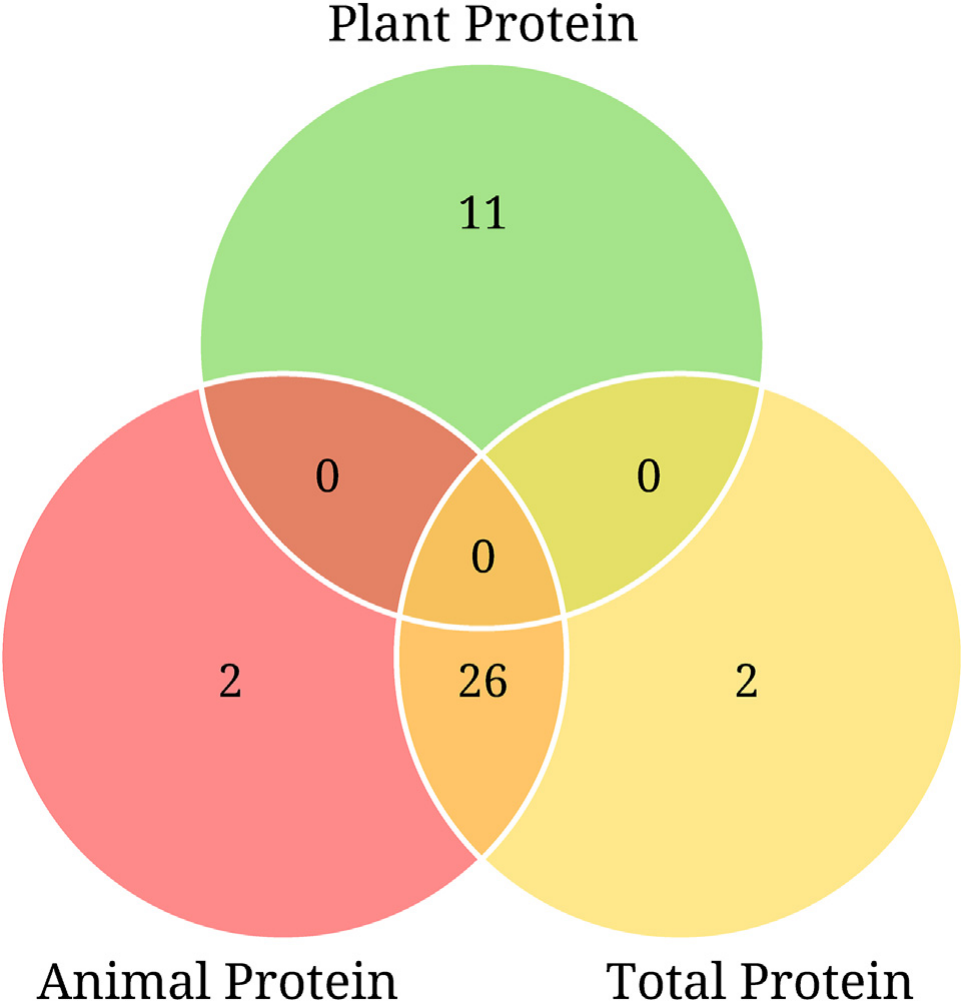
The number of unique and overlapping serum metabolites significantly associated with intake of total protein, animal protein, and plant protein in the Atherosclerosis Risk in Communities Study.

**FIGURE 2:**
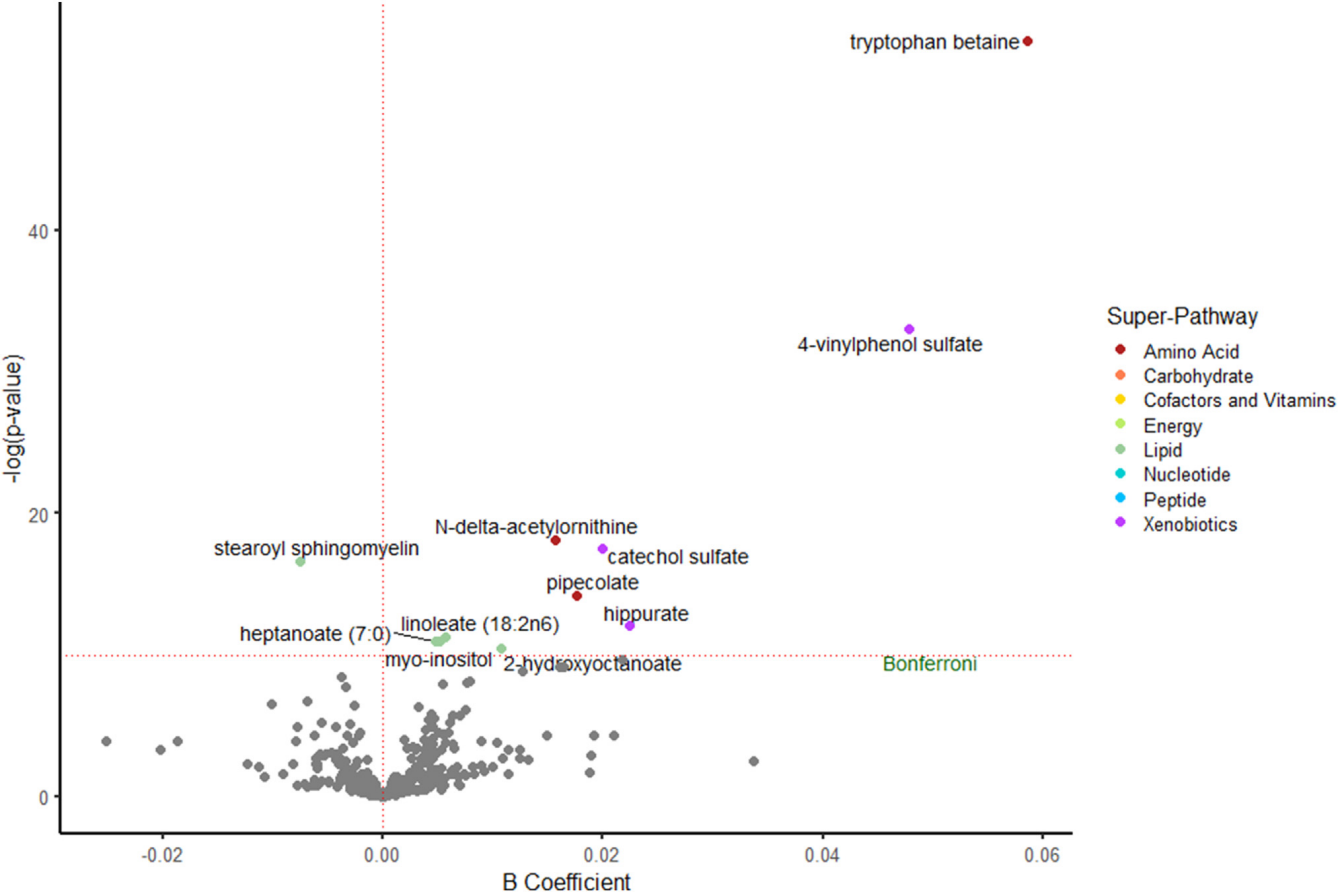
The associations of serum metabolites with intake of plant protein in the Atherosclerosis Risk in Communities (ARIC) study. Linear regression models adjusted for age, sex, race (in subgroup 2), study center (in subgroup 2), BMI, total energy intake, estimated glomerular filtration rate based on creatinine, smoking status, physical activity, education, alcohol consumption, total fruit intake, whole grains intake, and refined grains intake. The red-dashed horizontal line represents the statistical significance threshold after accounting for multiple comparisons using the Bonferroni method [*y* = −ln (0.05/(360 metabolites shared across both subgroups × 3 protein sources) = 9.98]. The red-dashed vertical line represents the null value of *β* = 0. Associations were meta-analyzed across the two subgroups using fixed-effects regression models.

There were 67 significant protein–metabolite associations in the primary analysis of 360 metabolites in both samples, and 41 unique metabolites were significantly associated with dietary protein intake (Figure 1). Plant protein was uniquely associated with 11 metabolites: tryptophan betaine, 4-vinylphenol sulfate, *N*-δ-acetylornithine, catechol sulfate, stearoyl sphingomyelin, pipecolate, hippurate, linoleate [18:2n-6 (ω-6)], heptanoate (7:0), myo-inositol, and 2-hydroxyoctanoate (Figure 2). Tryptophan betaine and 4-vinylphenol sulfate were the most strongly associated with plant protein. With the exception of stearoyl sphingomyelin, all these metabolites were positively associated with plant protein. The plant protein–related metabolites represented several metabolic superpathways: amino acids (*n* = 3), xenobiotics (*n* = 3), and lipids (*n* = 5) (Table 2). The most common subpathway represented among the plant protein–associated metabolites was benzoate metabolism (*n* = 3).

**TABLE 2:** Serum metabolites in both subgroups significantly associated with intake of plant protein in the ARIC study^1^

Metabolite	Superpathway	Subpathway	Meta-analyzed *β*	Meta-analyzed SE	Meta-analyzed *P*
Tryptophan betaine	Amino acid	Tryptophan metabolism	0.059	0.006	6.48 × 10^−24^
4-Vinylphenol sulfate	Xenobiotics	Benzoate metabolism	0.048	0.006	4.42 × 10^−15^
*N*-δ-acetylornithine	Amino acid	Urea cycle; arginine and proline metabolism	0.016	0.003	1.34 × 10^−8^
Catechol sulfate	Xenobiotics	Benzoate metabolism	0.020	0.004	2.41 × 10^−8^
Stearoyl sphingomyelin	Lipid	Sphingolipid metabolism	−0.008	0.001	5.82 × 10^−8^
Pipecolate	Amino acid	Lysine metabolism	0.018	0.004	6.72 × 10^−7^
Hippurate	Xenobiotics	Benzoate metabolism	0.022	0.005	5.90 × 10^−6^
Linoleate (18:2n-6)	Lipid	Polyunsaturated fatty acid (n-3 and n-6)	0.006	0.001	1.23 × 10^−5^
Heptanoate (7:0)	Lipid	Medium-chain fatty acid	0.005	0.001	1.71 × 10^−5^
Myo-inositol	Lipid	Inositol metabolism	0.005	0.001	1.72 × 10^−5^
2-Hydroxyoctanoate	Lipid	Fatty acid, monohydroxy	0.011	0.003	2.78 × 10^−5^

ARIC, Atherosclerosis Risk in Communities; SE, standard error.

1 Linear regression models adjusted for age, sex, race (in subgroup 2), study center (in subgroup 2), BMI, total energy intake, estimated glomerular filtration rate based on creatinine concentration, smoking status, physical activity, education, alcohol consumption, total fruit intake, whole grains intake, and refined grains intake. Bonferroni-adjusted *P* value = 0.05/(360 metabolites × 3 protein sources) = 4.63 × 10^−5^. Associations were meta-analyzed across the two subgroups using fixed-effects regression models.

**FIGURE 3:**
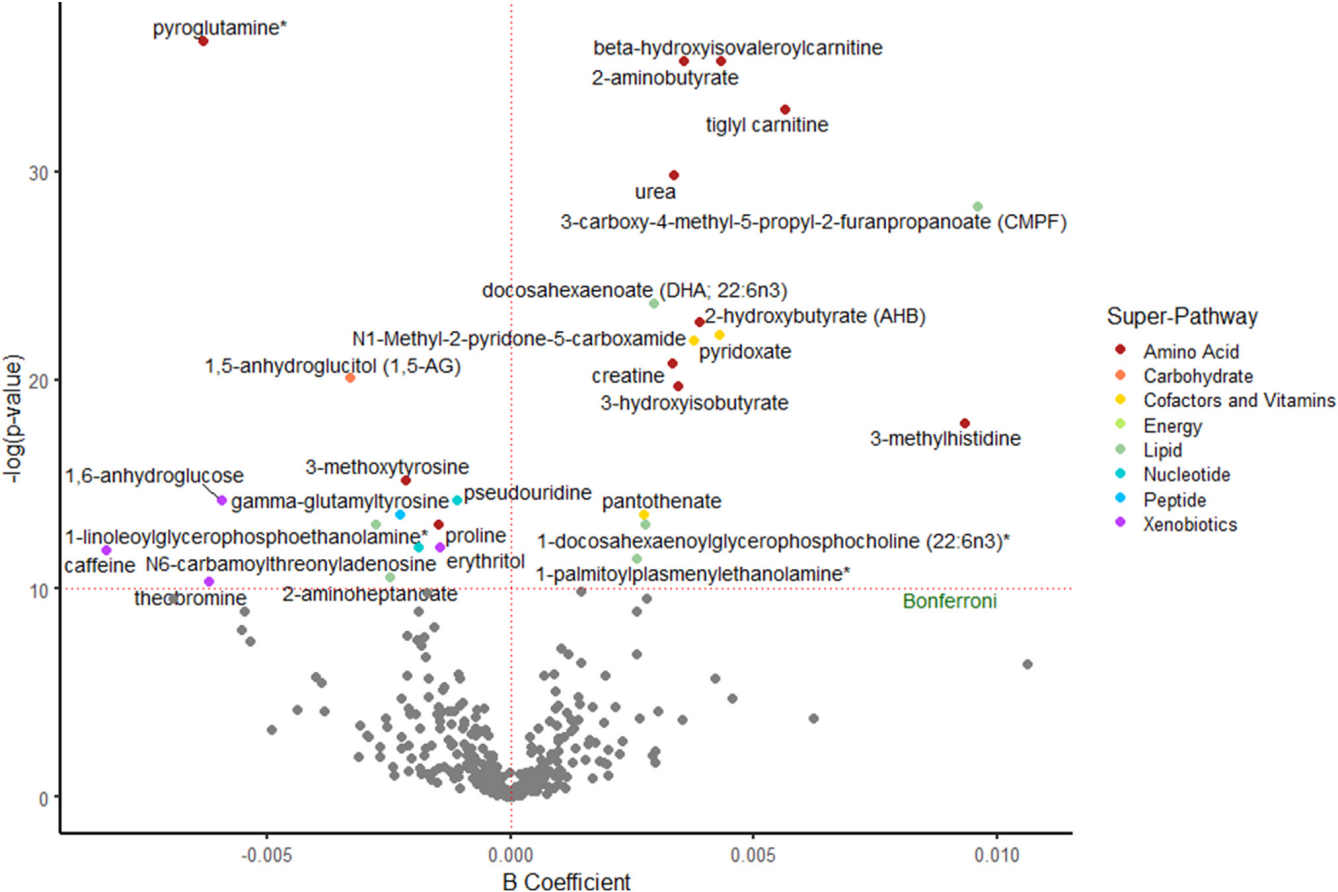
The associations of serum metabolites with intake of animal protein in the Atherosclerosis Risk in Communities (ARIC) study. Linear regression models adjusted for age, sex, race (in subgroup 2), study center (in subgroup 2), BMI, total energy intake, estimated glomerular filtration rate based on creatinine, smoking status, physical activity, education, alcohol consumption, total fruit intake, whole grains intake, and refined grains intake. The red-dashed horizontal line represents the statistical significance threshold after accounting for multiple comparisons using the Bonferroni method [*y* = −ln (0.05/(360 metabolites shared across both subgroups × 3 protein sources) = 9.98]. The red-dashed vertical line represents the null value of *β* = 0. Associations were meta-analyzed across the two subgroups using fixed-effects regression models.

There were 30 significant associations between metabolites and either total protein or animal protein, most of which (*n* = 26) were associated with both total protein and animal protein (Figure 1 and Table 3). The direction of the association was similar for these metabolites with total protein and animal protein. The 30 metabolites represented several metabolic superpathways: amino acids (*n* = 11), carbohydrates (*n* = 1), cofactors and vitamins (*n* = 3), lipids (*n* = 6), nucleotides (*n* = 2), peptides (*n* = 2), and xenobiotics (*n* = 5). 3-Carboxy-4-methyl-5-propyl-2-furanpropanoate (CMPF) and 3-methylhistidine were the most strongly associated with total protein and animal protein. The most common subpathways of metabolites that were significantly associated with animal protein were lysolipids (*n* = 3) and leucine, isoleucine, and valine metabolism (*n* = 3). Two metabolites (1-linoleoylglycerophosphoethanolamine and 2-aminoheptanoate) were uniquely associated with animal protein (Figure 3), and two metabolites (2-hydroxyisobutyrate and γ-glutamylphenylalanine) were uniquely associated with total protein (Supplemental Figure 2).

**TABLE 3:** Serum metabolites in both subgroups significantly associated with intake of total protein or animal protein in the ARIC study^1^

Metabolite	Superpathway	Subpathway	Animal protein			Total protein		
			Meta-analyzed *β*	Meta-analyzed SE	Meta-analyzed *P*	Meta-analyzed *β*	Meta-analyzed SE	Meta-analyzed *P*
Pyroglutamine∗	Amino acid	Glutamate metabolism	−0.006	0.001	1.75 × 10^−16^	−0.006	0.001	1.33 × 10^−16^
β-Hydroxyisovaleroylcarnitine	Amino acid	Leucine, isoleucine, and valine metabolism	0.004	0.001	4.54 × 10^−16^	0.004	0.001	1.09 × 10^−14^
2-Aminobutyrate	Amino acid	Methionine, cysteine, SAM, and taurine metabolism	0.004	0.000	4.66 × 10^−16^	0.004	0.000	4.01 × 10^−17^
Tiglyl carnitine	Amino acid	Leucine, isoleucine, and valine metabolism	0.006	0.001	4.60 × 10^−15^	0.006	0.001	4.67 × 10^−15^
Urea	Amino acid	Urea cycle; arginine and proline metabolism	0.003	0.000	1.11 × 10^−13^	0.003	0.000	6.60 × 10^−14^
3-Carboxy-4-methyl-5-propyl-2-furanpropanoate	Lipid	Fatty acid and dicarboxylate	0.010	0.001	4.88 × 10^−13^	0.010	0.001	9.15 × 10^−13^
Docosahexaenoate (22:6n-3)	Lipid	Polyunsaturated fatty acid (n-3 and n-6)	0.003	0.000	5.11 × 10^−11^	0.003	0.000	7.22 × 10^−11^
2-Hydroxybutyrate	Amino acid	Methionine, cysteine, SAM, and taurine metabolism	0.004	0.001	1.29 × 10^−10^	0.004	0.001	3.95 × 10^−11^
Pyridoxate	Cofactors and vitamins	Vitamin B-6 metabolism	0.004	0.001	2.36 × 10^−10^	0.005	0.001	5.10 × 10^−12^
*N*1-methyl-2-pyridone-5-carboxamide	Cofactors and vitamins	Nicotinate and nicotinamide metabolism	0.004	0.001	3.06 × 10^−10^	0.004	0.001	7.09 × 10^−11^
Creatine	Amino acid	Creatine metabolism	0.003	0.001	9.23 × 10^−10^	0.003	0.001	1.70 × 10^−9^
1,5-Anhydroglucitol	Carbohydrate	Glycolysis, gluconeogenesis, and pyruvate metabolism	−0.003	0.001	1.86 × 10^−9^	−0.003	0.001	1.16 × 10^−9^
3-Hydroxyisobutyrate	Amino acid	Leucine, isoleucine, and valine metabolism	0.003	0.001	2.73 × 10^−9^	0.004	0.001	1.12 × 10^−9^
3-Methylhistidine	Amino acid	Histidine metabolism	0.009	0.002	1.56 × 10^−8^	0.010	0.002	1.05 × 10^−9^
3-Methoxytyrosine	Amino acid	Phenylalanine and tyrosine metabolism	−0.002	0.000	2.47 × 10^−7^	−0.002	0.000	3.18 × 10^−7^
Pseudouridine	Nucleotide	Pyrimidine metabolism, uracil containing	−0.001	0.000	6.40 × 10^−7^	−0.001	0.000	1.62 × 10^−6^
1,6-Anhydroglucose	Xenobiotics	Food component/plant	−0.006	0.001	6.62 × 10^−7^	−0.006	0.001	8.71 × 10^−7^
γ-Glutamyltyrosine	Peptide	γ-Glutamyl amino acid	−0.002	0.000	1.25 × 10^−6^	−0.003	0.000	1.25 × 10^−7^
Pantothenate	Cofactors and vitamins	Pantothenate and CoA metabolism	0.003	0.001	1.26 × 10^−6^	0.003	0.001	3.94 × 10^−8^
1-Linoleoylglycerophosphoethanolamine∗	Lipid	Lysolipid	−0.003	0.001	2.06 × 10^−6^	NS	NS	NS
Proline	Amino acid	Urea cycle; arginine and proline metabolism	−0.001	0.000	2.12 × 10^−6^	−0.002	0.000	7.84 × 10^−7^
1-Docosahexaenoylglycerophosphocholine (22:6n-3)∗	Lipid	Lysolipid	0.003	0.001	2.13 × 10^−6^	0.003	0.001	1.59 × 10^−6^
*N*6-carbamoylthreonyladenosine	Nucleotide	Purine metabolism, adenine containing	−0.002	0.000	6.00 × 10^−6^	−0.002	0.000	9.44 × 10^−6^
Erythritol	Xenobiotics	Food component/plant	−0.001	0.000	6.29 × 10^−6^	−0.001	0.000	1.55 × 10^−5^
Caffeine	Xenobiotics	Xanthine metabolism	−0.008	0.002	6.93 × 10^−6^	−0.009	0.002	4.05 × 10^−6^
1-Palmitoylplasmenylethanolamine∗	Lipid	Lysolipid	0.003	0.001	1.05 × 10^−5^	0.002	0.001	2.46 × 10^−5^
2-Aminoheptanoate	Lipid	Fatty acid, amino acid	−0.003	0.001	2.69 × 10^−5^	NS	NS	NS
Theobromine	Xenobiotics	Xanthine metabolism	−0.006	0.001	3.23 × 10^−5^	−0.007	0.001	1.09 × 10^−5^
2-Hydroxyisobutyrate	Xenobiotics	Chemical	NS	NS	NS	0.002	0.000	1.49 × 10^−5^
γ-Glutamylphenylalanine	Peptide	γ-Glutamyl amino acid	NS	NS	NS	−0.002	0.000	1.67 × 10^−5^

ARIC, Atherosclerosis Risk in Communities; NS, not significant (*P* ≥ 4.63 × 10^−5^); SE, standard error.

1 Linear regression models adjusted for age, sex, race (in subgroup two), study center (in subgroup two), BMI, total energy intake, estimated glomerular filtration rate based on creatinine, smoking status, physical activity, education, alcohol consumption, total fruit intake, whole grains intake, and refined grains intake. Bonferroni-adjusted *P* value= 0.05/(360 metabolites × 3 protein sources) = 4.63 × 10^−5^. Associations were meta-analyzed across the two subgroups using fixed-effects regression models.

For the secondary analysis of metabolites available in only one subgroup, there were 17 significant protein–metabolite associations (total protein, *n* = 5; animal protein, *n* = 4; plant protein, *n* = 8) (Supplemental Table).

## Discussion

In 3914 middle-aged adults, we identified 41 serum metabolites significantly associated with dietary protein intake. Plant protein was significantly associated with seven metabolites (i.e., tryptophan betaine, 4-vinylphenol sulfate, catechol sulfate, hippurate, *N*-δ-acetylornithine, pipecolate, and linoleate) that have been previously associated with dietary sources of plant protein. Ten associations between animal protein and metabolites [i.e., pyroglutamine, creatine, phosphocholine (22:6), 3-methylhistidine, β-hydroxyisovaleroylcarnitine, pseudouridine, *N*6-carbamoylthreonyladenosine, CMPF, docosahexaenoate (DHA), and 1-docosahexaenoyl glycerophosphocholine (GPC)] were consistent with previous studies. Our untargeted approach yielded 24 promising new protein biomarkers, such as associations between plant protein and four lipids (heptanoate, myo-inositol, 2-hydroxyoctanoate, and stearoyl sphingomyelin). Our findings advance understanding of candidate biomarkers of dietary protein intake.

Our metabolomic findings were specific for plant protein and distinct from the animal protein–related metabolites. Tryptophan betaine and 4-vinylphenol sulfate were the most strongly associated metabolites with plant protein. Tryptophan betaine has been detected in legumes and is the precursor to indolylacrylic acid found in lentil seedlings [[25], [26], [27]]. Tryptophan betaine and 4-vinylphenol sulfate were associated with peanuts, a rich source of plant protein [28]. Plant protein was also associated with two xenobiotics, catechol sulfate and hippurate, involved in benzoate metabolism. Benzoic acid is naturally present in fruits and fermented products, and benzoate is an antimicrobial additive used in fruits and vegetables [29]. These two xenobiotics were inversely related to dietary acid load in a previous ARIC analysis [30]. Diets high in base-producing foods (e.g., fruits and vegetables) result in lower dietary acid load. Thus, our plant protein–metabolite findings are consistent with knowledge on food metabolism and previous metabolomic findings.

Plant protein was associated with two additional amino acids, *N*-δ-acetylornithine and pipecolate, and five lipids. *N*-δ-acetylornithine was quantified in oyster mushrooms, and pipecolate was identified in beans [31,32]. After dry bean consumption, serum levels of pipecolate were elevated in human and animal studies [33]. One of the lipids, linoleate, has been identified in canola and sunflower oils [34,35]. The other 4 lipids associated with plant protein (heptanoate, myo-inositol, 2-hydroxyoctanoate, and stearoyl sphingomyelin) have not been previously linked to dietary sources. Altogether, seven plant protein–related metabolites were consistent with previous studies, and four lipid associations were novel findings, which may serve as an impetus to better characterize the effect of dietary intake of plant protein on lipid metabolism.

Animal and total protein shared 26 of the 30 metabolite associations, which was unsurprising given that nearly three-quarters of total dietary protein intake came from animal protein sources, which is typical of United States diets [36]. In this study, pyroglutamine and creatine were associated with total protein and animal protein. These two metabolites have been previously associated with meat consumption [37]. In addition, creatine concentration was associated with animal protein intake in PREDIMED and with an animal protein diet pattern in the MASALA study [11,12]. The MASALA study also identified an association between the animal protein diet pattern and a lysophospholipid, 1-docosahexaenoyl-GPC (22:6), which was positively associated with animal protein in our study. We also found a significant association between animal protein and 3-methylhistidine. 3-Methylhistidine differentiated between animal protein and soy protein in a feeding trial [10]. Finally, we identified three metabolites (β-hydroxyisovaleroylcarnitine, tigyl carnitine, and 3-hydroxyisobutyrate) from the leucine, isoleucine, and valine metabolism subpathway, which were associated with animal protein. These essential amino acids are detected in high quantities in animal protein sources [38]. β-Hydroxyisovaleroylcarnitine was identified as one of the top differentiating metabolites between animal protein–based and soy protein–based diets in a feeding trial [10].

Our study identified significant associations between animal protein and metabolites found in animal protein. Pseudouridine and *N*6-carbamoylthreonyladenosine have been identified in cow’s milk [39]. Furan fatty acids such as furanpropanoate (CMPF) are related to the consumption of n-3 (ω-3) polyunsaturated fatty acid and were associated with fish and fish oils [[40], [41], [42], [43]]. It is encouraging that we observed significant and positive associations between animal protein and 2 n-3 (ω-3) polyunsaturated fatty acids—DHA and 1-docosahexaenoyl-GPC—and CMPF. In total, 10 metabolites (pyroglutamine, creatine, phosphocholine 22:6, 3-methylhistidine, β-hydroxyisovaleroylcarnitine, pseudouridine, *N*6-carbamoylthreonyladenosine, CMPF, DHA, and 1-docosahexaenoyl-GPC) associated with total and animal protein were consistent with previous metabolomic studies.

Our study had several limitations. Our data inherited biases from self-reported dietary information (e.g., recall bias, social desirability bias, and portion size misestimation), although the questionnaire had high reproducibility [16]. There is a need for additional biomarker discovery research as an alternative approach to dietary assessment through self-reporting. This study lays the groundwork for future feeding trials to validate our findings. Due to the observational study design, we cannot rule out the possibility of residual confounding, although it was minimized by administrating standardized questionnaires by trained interviewers and inclusion of multiple covariates in multivariable regression analyses. Biospecimens were stored for over two decades before metabolomic profiling. Degradation of compounds would be nondifferential by the level of dietary protein, thereby resulting in the attenuation of estimates. The stability of compounds in long-term stored specimens has been demonstrated by moderate correlation (Pearson correlation coefficients ≥0.65) between urea, glucose, and creatinine measured using standard clinical measures in 1989 compared with the levels of these compounds quantified using metabolomic profiling [44]. Metabolomic profiling was conducted in two subgroups at two time points. There was a high correlation (median Pearson correlation coefficient = 0.71) for 285 metabolites measured in 97 participants in 2010 and 2014, and our analyses were conducted separately within each subgroup and meta-analyzed [45]. Metabolites were measured in blood collected at one point in time. More work is needed to understand how the food metabolome changes over time.

This study has several strengths. We analyzed metabolomic data from a large, biracial cohort study that was geographically diverse. To our knowledge, this is the largest untargeted metabolomic study to report metabolites associated with dietary intake of plant protein [11] and one of the first studies to report metabolites associated with dietary intake of animal protein [[10], [11], [12]]. Our untargeted approach was advantageous in that it provided a comprehensive profile of the metabolome, allowing us to not only confirm previously observed findings but also to discover new markers of dietary protein intake. The metabolomic platform provided coverage of food-derived compounds labeled as xenobiotics, which enriched our findings as three xenobiotics (4-vinylphenol sulfate, catechol sulfate, and hippurate) were significantly associated with plant protein. We studied 2 subgroups from the ARIC study, which were demographically distinct, which allowed us to robustly assess the replicability of our findings.

In conclusion, we discovered 41 serum metabolites significantly associated with dietary protein in 3914 Black and White men and women. Seventeen of the 41 (41%) significant metabolites were consistent with prior metabolomic results. Thus, these metabolites are candidate markers of dietary protein intake. We also identified 24 new biomarkers of dietary protein, such as lipids related to plant protein intake. With external validation, these metabolites may eventually be used for objectively assessing dietary protein intake.

## Data Availability

The data described in the manuscript, code book, and analytic code will be made available on request pending application to the National Heart, Lung, and Blood Institute (NHLBI) Biologic Specimen and Data Repository Information Coordinating Center (BioLINCC) or to the Atherosclerosis Risk in Communities (ARIC) Study Publications Committee.

## Footnotes

Appendix A Supplementary data to this article can be found online at https://doi.org/10.1016/j.cdnut.2023.100067.
