# Supplementary material for: Metabolomics of Dietary Intake of Total, Animal, and Plant Protein: Results from the Atherosclerosis Risk in Communities (ARIC) Study
Source: Curr Dev Nutr. 2023 Mar 24;7(4):100067. doi: 10.1016/j.cdnut.2023.100067 (PMC10257224; doi:10.1016/j.cdnut.2023.100067)
Supplement: Multimedia component 1 [file mmc1.docx]

**Supplemental Figure 1.** Flow Chart of Study Participant Selection

ARIC Study Participants at visit 1

*n* = 15,792

Not assessed for eligibility:

Metabolomic data not available (*n* = 11,760)

Excluded (Total = 118; subgroup 1: *n* = 38, subgroup 2: *n* = 80):

Missing body mass index (subgroup 1: *n* = 1, subgroup 2: *n* = 3)

Missing total energy intake (subgroup 1: *n* = 0, subgroup 2: *n* = 59)

Missing or unknown smoking status (subgroup 1: *n* = 5, subgroup 2: *n* = 0)

Missing physical activity information (subgroup 1: *n* = 4, subgroup 2: *n* = 11)

Missing education status (subgroup 1: *n* = 5, subgroup 2: *n* = 0)

Missing alcohol intake (subgroup 1: *n* = 23, subgroup 2: *n* = 7)

Assessed for eligibility

*n =* 4,032

Included in analysis

*n =* 3,914

**Supplemental Figure 2.** Associations of Serum Metabolites with Intake of Total Protein in the ARIC Study^1,2^


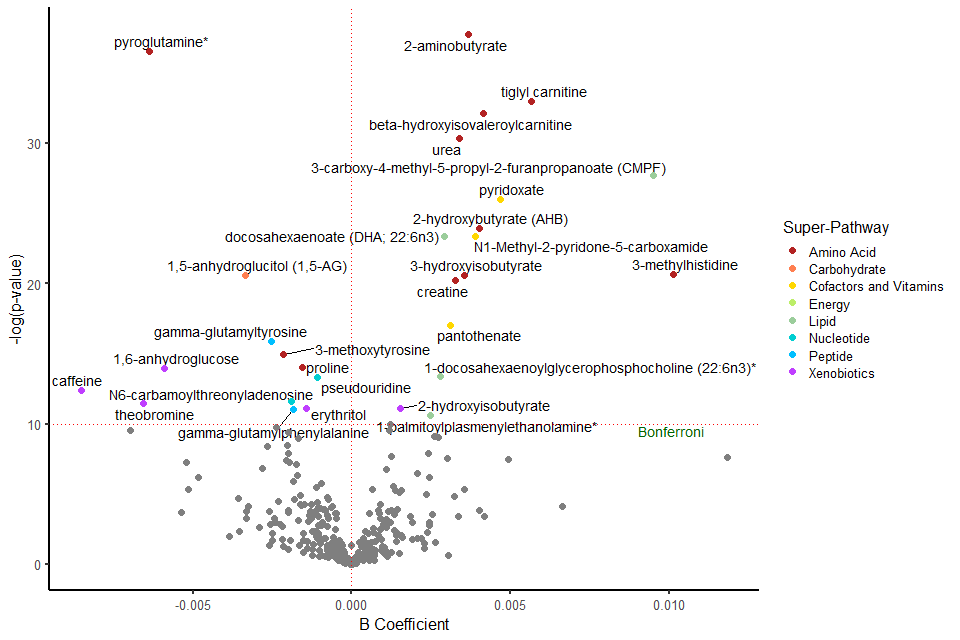


^1^ Linear regression models adjusted for age, sex, race (in subgroup 2), study center (in subgroup 2), body mass index, total energy intake, estimated glomerular filtration rate based on creatinine, smoking status, physical activity, education, alcohol consumption, total fruit intake, whole grains intake, and refined grains intake. The red dashed horizontal line represents the statistical significance threshold after accounting for multiple comparisons using the Bonferroni method [y = -ln (0.05/ (360 metabolites shared across both subgroups x 3 protein sources) = 9.98]. The red dashed vertical line represents the null value of β=0.

^2^ Associations were meta-analyzed across the two subgroups using fixed effects regression models.

Abbreviation: ARIC, Atherosclerosis Risk in Communities

**Supplemental Table 1.** Associations Between Serum Metabolites Available Only in Subgroup 2 and Dietary Intake of Protein in the ARIC Study^1^

| Metabolite | Superpathway | Subpathway | Protein Source Associated With | Beta | SE | P-Value |
| --- | --- | --- | --- | --- | --- | --- |
| guanidinosuccinate | Amino Acid | Guanidino and Acetamido Metabolism | Animal Protein | 0.017 | 0.003 | 5.69 × 10^-10^ |
| sulfate* | Xenobiotics | Chemical | Animal Protein | 0.001 | 0.000 | 3.21 × 10^-8^ |
| palmitoyl-linoleoyl-glycerophosphoinositol (1)* | Lipid | Lysolipid | Animal Protein | -0.004 | 0.001 | 1.23 × 10^-5^ |
| 2-docosapentaenoylglycerophosphocholine (22:5n3)* | Lipid | Lysolipid | Animal Protein | -0.018 | 0.004 | 4.37 × 10^-5^ |
| guanidinosuccinate | Amino Acid | Guanidino and Acetamido Metabolism | Total Protein | 0.018 | 0.003 | 1.60 × 10^-10^ |
| sulfate* | Xenobiotics | Chemical | Total Protein | 0.001 | 0.000 | 5.70 × 10^-8^ |
| palmitoyl-linoleoyl-glycerophosphoinositol (1)* | Lipid | Lysolipid | Total Protein | -0.004 | 0.001 | 6.23 × 10^-6^ |
| 2-docosapentaenoylglycerophosphocholine (22:5n3)* | Lipid | Lysolipid | Total Protein | -0.020 | 0.005 | 9.43 × 10^-6^ |
| pyridoxal | Cofactors and Vitamins | Vitamin B6 Metabolism | Total Protein | 0.010 | 0.002 | 3.36 × 10^-5^ |
| methylsuccinate | Amino Acid | Leucine, Isoleucine and Valine Metabolism | Plant Protein | 0.008 | 0.002 | 4.50 × 10^-6^ |
| trigonelline (N'-methylnicotinate) | Cofactors and Vitamins | Nicotinate and Nicotinamide Metabolism | Plant Protein | 0.038 | 0.009 | 1.26 × 10^-5^ |
| arachidate (20:0) | Lipid | Long Chain Fatty Acid | Plant Protein | 0.007 | 0.001 | 1.35 × 10^-5^ |
| 1-arachidoylglycerophosphocholine (20:0) | Lipid | Lysolipid | Plant Protein | 0.015 | 0.004 | 1.66 × 10^-5^ |
| myristoyl sphingomyelin* | Lipid | Sphingolipid Metabolism | Plant Protein | -0.007 | 0.002 | 3.09 × 10^-5^ |
| N-(2-furoyl)glycine | Xenobiotics | Food Component/Plant | Plant Protein | 0.024 | 0.006 | 3.33 × 10^-5^ |
| beta-sitosterol | Lipid | Sterol | Plant Protein | 0.027 | 0.007 | 3.75 × 10^-5^ |
| 4-acetylphenol sulfate | Xenobiotics | Drug | Plant Protein | 0.030 | 0.007 | 4.47 × 10^-5^ |

^1^Linear regression models adjusted for age, sex, race, study center, body mass index, total energy intake, estimated glomerular filtration rate based on creatinine, smoking status, physical activity, education, alcohol consumption, total fruit intake, whole grains intake, and refined grains intake. Bonferroni-adjusted p-value for subgroup 2= 0.05/ (365 metabolites analyzed in subgroup 2 only x 3 protein sources) = 4.56 × 10^-5^.

Abbreviations: ARIC, Atherosclerosis Risk in Communities; SE, standard error
